# Effect of Pressure biofeedback training on deep cervical flexors endurance in patients with mechanical neck pain: A randomized controlled trial

**DOI:** 10.12669/pjms.37.2.2343

**Published:** 2021

**Authors:** Rabia Ashfaq, Huma Riaz

**Affiliations:** 1 Dr. Rabia Ashfaq, DPT, MS (OMPT). Physical Therapist, Department of Rehabilitation sciences, Riphah International University, Rawalpindi, Pakistan; 2 Huma Riaz, PHD*(Rehab Sciences), PP-DPT, PGD (PE&TM), Bs.PT Associate Professor/ Head of Department DPT, Riphah College of Rehabilitation & Allied Health Sciences, Islamabad, Pakistan

**Keywords:** Mechanical neck pain, Craniocervical flexion training, deep cervical flexors, pressure biofeedback, endurance

## Abstract

**Objective:**

To compare the effects of Cranio cervical flexion training with and without pressure biofeedback on deep cervical muscular endurance in patients with mechanical chronic neck pain.

**Methods:**

A randomized control trial was conducted at Railway General Hospital Rawalpindi, from May to December 2019. It consisted of thirty participants with the age ranging from 25 to 40 years, and having chronic mechanical neck pain. The participants were randomly allocated into two groups Group-A received Craniocervical flexion training with pressure biofeedback and Group-B received Craniocervical flexion training without pressure biofeedback. The intervention was applied for four weeks (3 sessions per week). Assessments were taken at Pre, Post intervention and after six weeks of follow up. Data analysis was done using SPSS-21 version.

**Results:**

The mean age of Group-A and Group-B was 29.40±3.08 and 31.33±4.95 respectively. Between-group analyses has shown statistically and clinically significant improvement in Group-A regarding deep neck muscles endurance (p<0.05). Whereas within group analysis of both groups A & B showed a statistical and clinically significant difference (p=0.00) for deep neck muscles endurance.

**Conclusions:**

Cranio-cervical flexion training with Pressure Biofeedback has proven to be more effective in improving endurance of deep cervical flexors in patients with mechanical neck pain.

## INTRODUCTION

Neck pain affects approximately 70% of people at some point in their lifetime.^1^ Gender wise distribution is women (43%) and men (30%).^2^ Mechanical neck pain is described by IASP as the nonspecific pain experienced posteriorly to the neck which originates from the superior nuchal line and extends to the first thoracic spinous process.^3^ In Asia 1-year point prevalence of neck pain is 13%.^4^ Chronic Mechanical Neck pain is associated with multiple factors such as sprain and strain of the muscles and ligaments of neck, repetitive movements, extended durations of same posture, poor workstation design, genetic predisposition, decreased strength and endurance of cervical muscles.^5^,^6^ It results in significant use of medication, work related absenteeism, impaired performance and poor quality of life. Neck pain is considered to be a problem which causes subsequent personal and financial loss.^7^

Maintenance of proper posture is found to be an important factor in management of neck pain.^8^ Deep cervical flexors (DCF) play a vital role in maintenance of posture control along with providing the stability to the cervical region. DCF get weakened during the chronic neck pain which results in hyperactivity of superficial neck muscles. This continuous imbalance between the superficial and deep flexor muscles contribute to the loss of correct lordotic alignment leading to various cervical impairments.^9^ Chronic neck pain also impairs neuromuscular coordination between superficial and deep muscles, which necessitates DCF training as an integral part of rehabilitation. Blomgren J et al. (2018) mentioned in a systematic review that DCF is low load, without resistance motor training program guided by feedback of inflated pressure sensor.^10^ Tsiringakis G et al (2020) recently concluded in a systematic review that it is preferable and more effective to induce motor control training of DCF with pressure biofeedback for neck pain and disability rather than only strength endurance training of cervical muscles.^11^

Previous studies have been revolving around therapeutic measures of superficial cervical muscles in patients with mechanical neck pain whereas scant amount of studies have been conducted on deep neck flexors training for alleviation of neck pain and endurance.^12^ There is emerging evidence that DCF training with pressure biofeedback is effective for pain reduction and endurance training.^13^,^14^ So, the current study intended to put through the benefits of deep cervical flexor training with pressure biofeedback for treatment of cervical neck pain patients which is hypothesized to contribute effectively in DCF endurance. The specific objectives of this study were to determine the effects of cranio-cervical flexion training with pressure biofeedback on pain and endurance in patients with mechanical neck pain.

## METHODS

This randomized control trail was conducted in physiotherapy department of Railway General Hospital Rawalpindi after obtaining ethical approval from research ethical committee of Riphah College of Rehabilitation & Allied Health Sciences. (Ref. No. Riphah/RCRS/REC/00559) from May 2019 to December 2019. The present study trial was registered at www.ClinicalTrails.gov with registry number NCT04173143. Participants selected through non-probability purposive sampling technique. Sample size was calculated by Openepi version 3 software, using NPRS variable values, level of significance was kept 0.5 and power of the study was 0.80. The calculated sample size was 24 but it was extrapolated to 30 to handle drop outs.^7^ After screening, 30 patients with age 25-40 years, having chronic neck pain for more than three months, with Numeric Pain Rating Score (NPRS) greater than three were selected. Patients having history of cervical trauma, any Spinal cord deformities, significant neurological deficit, any postural deformity, pain from non-musculoskeletal causes, or had a history of malignancy, current pregnancy were excluded from the study. They were randomly allocated in two groups by sealed envelope method. Informed consent was taken from all the participants and baseline data as well as measurements were taken. Patient Performa included demographic information, neck pain history, NPRS and endurance scores in mmHg as well as Hold time in seconds.

The primary outcome measure of the study was endurance of DCF which was measured by Deep Neck Flexor (DNF) Endurance Test and *Craniocervical Flexion Test (CCFT)*. Inter rater reliability (ICC) of DNF Endurance Test (hold time in sec) is 0.82-.91.^15^ It is performed with the patient in hook lying position. The patient was instructed to maximally retract the chin and lift the head and neck until the head was round about 2.5cm (1 inch) above the couch. To calculate the length of time (in seconds) a stopwatch was used. The test ended either the line along patient neck started to isolate or the patient head touches the examiner hand for more than one second.^16^ The ICC for the *CCFT(mmHg)* range from 0.81 to 0.98.^17^ It is performed in supine crook position. The neck was aligned in a neutral position. The pressure biofeedback unit was centered just below the occiput between the plinth and the back of the neck and inflated to a baseline of 20 mmHg. Each subject was asked to gently and slowly execute the head nodding action (as if they said yes) at five different levels of pressure (22, 24, 26, 28 and 30 mmHg) and to maintain each level for 10 seconds There was a 30-second rest period between each level. The test protocol ended when the subject was unable to hold the same pressure level for 10 sec or reached a peak level of 30mmHg.^18^,^19^

After initial assessment all the subjects were randomized into two groups. Group-A received Craniocervical flexion training with pressure biofeedback, Patients were asked to lie in supine hook lying position. Pressure biofeedback unit’s inflated to 20mmHg and was positioned behind the neck and the dial which was connected to the pressure sensor, was given to the patient. Patients were advised to perform Craniocervical flexion action to progressively target (reach the incremental targets) and hold the 5 pressure levels for 10 second between 22 mm Hg and 30 mmHg. A 2-minute second rest period was provided between each level. Session was performed thrice in a week. Each session was given for approximately 20 minutes.^16^,^20^ Group-B received Craniocervical flexion training without pressure biofeedback. The patient lied in crook lying position. The patient was asked to keep the chin maximally retracted and raise the head and neck approximately two to five cm (one inch) above the couch. Patients were instructed to perform 10 repetitions for a hold of 20 seconds initially, increasing it by 10 seconds progressively. The entire session had a maximum of 4 sets and should perform 3 times in a week.^16^

The data was analyzed using SPSS Version 21. Shapiro Wilk test was applied to check normality of data. Parametric tests were applied on the data for p-value > 0.05 and non-Parametric test were applied on the data for p-value < 0.05.

## RESULTS

The total 30 number of participants were analyzed (experimental = 15, control = 15), the mean age of Group-A was 29.40±3.08 years, whereas for Group-B it was 31.33±4.95 years. The mean BMI of Group-A was 22.18±4.01, whereas for Group-B it was 23.47±2.23. Among total individuals, 20(%) were females and 10(%) were males in both Groups.

At pre-test level NPRS and endurance scores both in (mmHg) and (hold time in sec) were recorded. Second assessment was done for the said outcome measures after four weeks of intervention by the physical therapist. Patients followed home exercise interventions for further two weeks and then final measurements were taken after 6 weeks follow up (FU)

Group analyses, in which comparison was done using independent T Test. is shown in Table-I. After treatment, there was significant improvement of pain and endurance scores measured in (mmHg) and (hold time in sec) in Group-A at both 4^th^ week (p<0.05) as well as at Follow up (p<0.05).

**Table-I:** Between Group Analysis.

*Variables*	*n*	*TP*	*Groups*	*Mean ± SD (Degree)*	*P value*
NPRS	15	Pre	Group-A	6.33±1.11	1.00
			Group-B	6.33±1.34	
		Post	Group-A	1.67±0.89	<0.00
			Group-B	2.93±0.79	
		FU	Group-A	0.73±0.45	<0.00
			Group-B	2.20±0.67	
Endurance (mmHg)	15	pre	Group-A	24.53±1.92	0.62
			Group-B	23.47±0.91	
		post	Group-A	28.26±1.67	<0.00
			Group-B	24.27±1.27	
		FU	Group-A	29.60±1.12	<0.00
			Group-B	25.06±1.27	
Endurance (hold time in sec)	15	Pre	Group-A	13.47±4.94	1.00
			Group-B	13.46±4.94	
		Post	Group-A	28.00±3.46	0.013
			Group-B	24.00±4.67	
		FU	Group-A	30.20±1.47	0.032
			Group-B	28.00±3.46	

Results of within Group-A analysis are shown in Table-II. Friedman test was conducted for variables of pain and Endurance (Hold time in sec). Whereas for variable i.e. endurance (mmHg) one way repeated measure ANOVA test was applied. Likewise, Table-III shows within Group-B analysis. Friedman test was conducted for variables of pain whereas for Endurance (Hold time in sec) and (mmHg) one-way repeated measure ANOVA test was applied. Within group analysis result has found statistically significant improvement in both group (P<0.00) for both variables at 4^th^ week (p<0.05) as well as at Follow up (p<0.05).

**Table-II:** Within Group-A Analysis.

*Variables*	*TP Md MR P*	*n*	*Mean+SD (Degree)*	*Md (IQR)*	*MR*	*P value*
NPRS	Pre	15	6.33±1.11	6.00(1)	3.00	<0.00
	Post		1.67±0.89	2.00(1)	1.87	
	FU		0.73±0.45	1.00(1)	1.13	
Endurance (mmHg)	pre	15	24.53±1.92	-	-	<0.00
	post		28.26±1.67	-	-	
	FU		29.60±1.12	-	-	
Endurance (hold time in sec)	pre	15	13.46±4.94	14.00(11)	1.00	<0.00
	post		28.00±3.46	29.00(4)	2.20	
	FU		30.20±1.47	30.00(2)	2.80	

**Table-III:** Within Group-B Analysis.

*Variables*	*TP*	*n*	*Mean ± SD (degree)*	*Md (IQR)*	*MR*	*P value*
NPRS	Pre	15	6.33±1.34	6.00(2)	3.00	<0.00
	Post		2.93±0.79	3.00(2)	1.87	
	FU		2.20±0.676	2.00(1)	1.13	
Endurance (mmHg)	pre	15	23.46±0.91	-	-	<0.01
	post		24.26±1.27	-	-	
	FU		25.06±1.27	-	-	
Endurance (hold time in sec)	pre	15	13.46±4.94	-	-	<0.00
	post		24.00±4.67	-	-	
	FU		28.00±3.46	-	-	

## DISCUSSION

Neck pain is commonly prevalent chronic disorder with increasing economic burden to the society. A functional contributing factor to chronic mechanical neck pain may be the altered motor function of the cervical spine and its related micro and macro damage.^21^ The restoration of muscle functions therefore becomes an integral part of neck pain treatment.^10^ Existing literature inform us about measures to restore length and strength of superficial neck flexors. Using pressure biofeedback as measurement and therapeutic tool for muscle training is a novel and objective method. So far, fewer studies are comparing the deep cervical flexor training with and without pressure biofeedback. This study was designed with aim to determine the effects of Cranio cervical flexion training with and without pressure biofeedback on neck pain and DNF endurance.

The present study findings indicated that pressure biofeedback training is more efficient to improve neck pain. A similar study has been conducted by M Karthi et al. (2019) on efficacy of endurance training on deep cervical flexor muscles using pressure feedback in mechanical neck pain. They have concluded that Deep Cervical Flexor Training with Visual Pressure Biofeedback was significantly effective (p<0.005) for reduction in neck pain than the conventional training.^10^ Kim JY et al. (2016) conducted a study on Clinical effects of deep cervical flexor muscle activation in patients with chronic neck pain. Reported findings suggest that pressure biofeedback for deep cervical flexor muscles training gave a better improvement in neck pain (p<0.05) than general strengthening exercises after four and eight weeks training.^9^ Superiority of the deep neck flexor exercise in pain outcome compared to isometric, stretching, and scapulothoracic exercises has been established in another randomized clinical trial.^22^ So, the existing literature support our current study findings in terms of effectiveness of DCF endurance training with pressure biofeedback as compared to conventional therapeutic exercise approaches.

It is evident from literature that poor endurance of neck muscles is considered a risk factor for mechanical neck pain. So, interventions targeted for its improvement given more emphasis in neck pain management protocols. The results of the present study have showed that individuals receiving Cranio cervical flexion training with pressure biofeedback unit improved more in deep neck flexor endurance measured (both in mmHg and hold in sec). These results are supported by M Karthi et al on efficacy of endurance training on deep cervical flexor muscles using pressure feedback in mechanical neck pain. Between-group and within the group analysis showed a statistical and clinically significant difference in terms of endurance.^10^ Another recent research finding goes in line with the current study findings. They provided evidence that DCFs training with pressure biofeedback was more effective than traditional physical therapy for improving neck proprioception, pain, muscle strength and dizziness in patients with cervical spondylosis.^23^ Contrary to current study, an RCT conducted by Al-Harbi et al. (2017) has stated superior effects of deep cervical flexor training without pressure biofeedback unit. But they have treated the other group with electrotherapeutic modalities in sufferers of neck pain due to overuse of smart phones.^24^

Due to practice pattern and gender based ethical concerns of clinical site, less male patients were recruited & male to female ratio was therefore 1:3. Further, It was researcher’s concern that the postures of study participants observed in a photographic analysis might not be true reflection of the one adopted while working. This study has provided a useful insight and addition in treatment protocol of mechanical neck pain using a valid intervention technique. The study should be done with larger sample size and by recruiting equal number of male patients. To improve the internal validity of the study, assessor blinding should be followed. Long term effects of interventions are needed to be followed.

## CONCLUSION

Cranio-cervical flexion training with pressure biofeedback proves to be more clinically effective in terms of neck pain and endurance. So, this specific, easy to use intervention is recommended to be part of routine physical therapy protocol for patients with chronic mechanical neck pain.

### Author’s Contribution:

**HR & RA:** conceived, designed and did statistical analysis & editing of manuscript

**RA & HR:** did data collection and manuscript writing

**RA & HR:** did data analysis and interpretation, accountable for integrity of work.

**HR:** did critical review, rechecked information accuracy and final approval of manuscript.
